# The Episodic Man: How a Psychological Biography of Donald J. Trump Casts New Light on Empirical Research Into Narrative Identity

**DOI:** 10.5964/ejop.4719

**Published:** 2021-08-31

**Authors:** Dan P. McAdams

**Affiliations:** 1 Department of Psychology, Northwestern University, Evanston, IL, USA; Nelson Mandela University, Port Elizabeth, South Africa

**Keywords:** psychological biography, narrative identity, Donald Trump, autobiographical memory, autobiographical reasoning, personality psychology

## Abstract

People make meaning through life narrative. The central thesis of my book-length psychological biography of Donald Trump is that the 45th president of the United States defied this general meaning-making tendency and epitomized instead the episodic man. Like no other president in modern history, Trump seems to be nearly devoid of a narrative identity, which is an internalized and evolving story of the self that reconstructs the personal past and imagines the future in order to provide life with temporal continuity and meaning. Instead, Trump has always lived in the emotionally vivid moment (episode), fighting to win each moment, moment by discrete moment. Seeing him through the lens of the episodic man helps to explain many puzzling features of Donald Trump’s personality, from his charismatic effect on millions of Americans to his penchant for lying and malice. Importantly, the analysis of Trump’s episodic nature informs the scientific study of narrative identity and meaning making more generally, suggesting that people vary not only with respect to the kinds of stories they create for their lives but also with respect to the extent to which they construe life in narrative terms. Therefore, the analysis of Trump illustrates the potentially reciprocal relationship between the idiographic case and the nomothetic effort to develop and evaluate more general scientific hypotheses.

Going back to Sigmund Freud’s famous case of Anna O. (Breuer & Freud, 1895/1955), empirical psychologists have mined biographical case studies for insights into general psychological processes. Allport (1937) argued that the science of personality psychology should ideally blend idiographic studies of particular individual lives with nomothetic surveys and experiments aimed at examining broad propositions as they apply to many, if not all, lives. In that they focus intensively on the individual life of a noteworthy person, psychological biographies are idiographic investigations *par excellence* (Mayer & Kovary, 2019; Schultz, 2005). In most instances, the primary aim of a psychological biography is to explain the life of the particular person chosen for scrutiny, in all its specificity and uniqueness. At the same time, specific interpretations of a single life may sometimes suggest new ideas for understanding other lives, as well. Idiographic psychological biographies may generate insights that can be pursued in nomothetic, hypothesis-testing research (McAdams & West, 1997). The insights may help to build psychological theory more generally, and to advance psychological science.

In what follows, I briefly describe how writing a psychological biography of Donald J. Trump produced insights that may generalize well beyond his unique individual case. In *The Strange Case of Donald J. Trump: A Psychological Reckoning* (McAdams, 2020), I analyzed Trump’s dispositional personality traits, his values and goals, his narcissistic personal agenda, his relationships with significant people in his life, his authoritarian sentiments, and the emotional grip he exerted on millions of Americans during the 4 years of his presidency (2017–2021). In so doing, I drew liberally from theory and research in personality, developmental, social, and political psychology, as well as cognitive science and evolutionary biology (see also McAdams, 2016, 2017). What gradually emerged, however, as the central thesis of the book seemed to defy at least one basic assumption in the psychological research literature. The assumption is that people create *narrative identities* for their lives during their adult years—internalized and evolving stories that explain who they are and how they have come to be the unique persons they are becoming (McAdams, 1985; McAdams & McLean, 2013). The research assumes that people make meaning in their lives through constructing integrative stories of the self.

But not Donald Trump. What eventually emerged as the central thesis of my 300-page psychological biography is that Donald Trump *simply has no narrative identity*. He lives outside of time and narrative, like no other person I have ever encountered. He is *the episodic man*—living forever in the combative moment, striving to win each moment, moment by moment, episode by discrete episode. As documented exhaustively in McAdams (2020), the episodes do not add up; they do not form a narrative arc in his mind, even if other people can see a story there. The strange case of Donald Trump calls into question a fundamental idea that has guided the research literature on narrative identity ever since the topic surfaced in personality and developmental psychology in the 1980s (McAdams, 1985). As such, this particular idiographic case study suggests new ways to think about narrative identity and new empirical leads to pursue in nomothetic research on the stories people create to make meaning in their lives. The primary intent of the current paper, then, is to show how a psychological biography of one prominent man has had some impact on how psychological scientists think about people more generally. The qualitative case study of a single life can suggest new ideas for quantitative research examining many different lives.

## Meaning and Narrative

How do people find *meaning* in life? How do people create lives deemed to be *meaningful*? Philosophers, psychologists, and laypeople offer many different answers to these questions (e.g., Markman, Proulx, & Lindberg, 2013). Nonetheless, a common theme in contemporary writing, both in the humanities and the social sciences, is that human beings make meaning through stories (Altmaeir, 2017; Bruner, 1990; Ricouer, 1984). According to Schechtman’s (1996) philosophical perspective, people apprehend their lives as ongoing narratives, situating themselves as protagonists in the middle of an unfolding plot. Lives have meaning to the extent that protagonists can step back from the fray to operate as authors of their lives, construing pattern and coherence in the narrative flow and communicating what they construe to other people, who aim to connect their own stories to what they hear. As people share with each other narrative accounts of their own lived experience, they implicitly evaluate what they hear and observe. Is the story understandable? Does it make sense? Is it a good story? Is it significant? Does it convey lived experience in a way that connects to my own life? Does it hold meaning for me?

Storytelling is a human universal. Whether conveyed in novels, movies, mime, or dance, people tell stories the world over. Humans tell stories for many different purposes—to entertain each other, to convey important social information, to offer instruction, to simulate experience, to gossip, to worship, and sometimes simply to fill the time, to alleviate the boredom or spice up the daily routine. One highly influential line of thinking in linguistic anthropology suggests that human language itself evolved primarily for the purpose of telling stories (Dor, 2015).

In all human cultures, children begin to tell stories about their own experience almost as soon as they begin to speak (Fivush, 2011). These stories ultimately shape the development of their own autobiographical memory and their sense of self. In childhood, narrative accounts of lived experience become attached to the self-concept, as little stories about things that happened to *me*—*my* stories. In adolescence and early adulthood, the little stories begin to amalgamate and rearrange themselves into a larger narrative about who I am, how I came to be, and where my life may be going (Habermas & Bluck, 2000). The larger narrative—evolving over time as it assimilates new experiences and reinterprets the remembered past in light of future goals—is a person’s narrative identity. Adults move through life, therefore, with a storied understanding of the reconstructed past and the imagined future. They feel that they are living within the story (as a protagonist) and constructing the story as they live in it (as a narrator, or author). The life story they construct, encompassing many different scenes and plotlines, is part and parcel of *who they are* (McAdams, 1985), fundamentally so. As such, it is central to what Erik Erikson (1963) described in more general terms as the “identity” (sometimes called “ego identity”) that people begin to apprehend and construct in their adolescent years. A person’s narrative identity, then, provides life with the very same attributes that Erikson (1963) originally ascribed to identity itself. Those include a sense of knowing who I am and how I came to be, feeling sameness and continuity over time, situating the self within a recognizable niche in the adult world, and experiencing life as more-or-less unified (across different roles), purposeful (goal-directed, self-determined), and meaningful (coherent, significant).

Over the past two decades, researchers have conducted hundreds of empirical studies on narrative identity, refining and articulating this influential psychological construct as it has evolved in the fields of personality, developmental, social, and cultural psychology, and in cognitive science (for reviews, see Adler, Lodi-Smith, Philippe, & Houle, 2016; McAdams, 2021; McAdams & McLean, 2013). Among the most important findings in the empirical literature on narrative identity are these:

- The emergence of narrative identity in the adolescent and early adult years coincides with and depends upon the development of *autobiographical reasoning* skills. Through autobiographical reasoning, people inductively derive semantic meanings regarding the self from the memories of episodic events (Habermas & Bluck, 2000). Autobiographical reasoning becomes more sophisticated and integrative as people move into the midlife years (Pasupathi & Mansour, 2006). In later life, autobiographical reasoning skills may become less differentiated and analytical, and they may rely more on the tendency to simplify and soften lived experience, often resulting in the accentuation of positive emotional themes (Baddeley & Singer, 2007). The developmental trend reflects what memory researchers describe as the *positivity effect* of aging (Carstensen & Mikels, 2005).
- Narrative identity develops gradually over time through a transactional and recursive interpersonal process. People create stories about events in their lives, tell those stories to others, monitor the reactions to their narrative performances, change their stories in response to those reactions, interpret new events in terms of past stories, draw on new events to change their stories of the self, tell those new stories to others, monitor feedback, and on and on. Selves create stories, which in turn create new selves, as narrative identity evolves over time (McLean, Pasupathi, & Pals, 2007).
- Narrative identities that prioritize themes of agency, belongingness, and personal redemption tend be strongly associated with psychological well-being, mental health, and a generative engagement with society in the adult years (Adler et al., 2015; McAdams & Guo, 2015). Individual differences in the salience of certain content themes in narrative identity predict well-being above and beyond the statistical effects shown for positive and negative personality traits, such as extraversion and neuroticism (Adler et al., 2016). By contrast, narrative identities that depict vicious cycles and contaminated plots tend to be associated with depression, anxiety, and other negative life outcomes (Adler, Kissel, & McAdams, 2006).
- Master cultural narratives shape the development of narrative identity (McLean & Syed, 2015). People draw from a culture’s storehouse of favored images, plotlines, characters, and narrative themes in constructing their own life stories. Whereas these cultural resources provide invaluable models for narrating life, they can also exert hegemonic effects that constrain human potential. As such, master cultural narratives reflect gender norms, class bias, and power inequities in society (Hammack, 2008).

A broad assumption in this research literature is that articulating a narrative identity is a normative developmental process in nearly all human lives. With the exception of people suffering from serious mental illnesses (e.g., schizophrenia, autism) or cognitive disabilities (e.g., dementia) that create profound disturbances in selfhood, nearly all human beings are expected to situate their lives within a temporal framework. In so doing, they are expected to create, and to communicate to others, internal life stories that make sense of the personal past and the anticipated future. In short, people are expected to “have” a narrative identity, even if each particular narrative identity is unique, differing from others in terms of structure, content, and other psychologically important features—and even if every culture provides its own specific rules and norms for how to make narrative sense of a life.

## The Strange Case of Donald J. Trump

In the Spring of 2016, an editor from *The Atlantic* magazine asked me to write an extended essay on the personality of Donald J. Trump, who was seeking the Republican nomination for President of the United States. He suggested I follow the model for an evidence-based, scientifically grounded psychological biography that I used in a previous study of George W. Bush, the 43rd President of the United States (McAdams, 2011). In that book, I examined Bush’s life and personality from the psychological standpoints of 1) the *social actor* (the dispositional personality traits and salient roles that characterized Bush’s emotional trends and daily behavior—his socio-emotional style), 2) the *motivated agent* (the goals, values, and infrastructure of belief that shaped Bush’s motivational agenda in life—what he wanted and valued), and 3) the *autobiographical author* (Bush’s narrative identity, as it developed from young adulthood through midlife—how he made meaning in his life). The tripartite model of actor/agent/author is today a highly influential framework for organizing theory and research in the field of personality psychology and in the study of life-span personality development (McAdams, 2015; McAdams & Olson, 2010).

The resultant essay on Trump (McAdams, 2016) focused on 1) the dispositional traits of (high) extraversion and (low) agreeableness that mainly characterize his daily behavior and 2) the supremely narcissistic motivational agenda Trump pursues, along with the authoritarian values that support his narcissism. But I struggled to find evidence for the third layer of personality development. An initial perusal of the biographical record on Trump provided few insights on narrative identity. In sum, I found it to be relatively easy to characterize Trump’s personality from the standpoints of the social actor and the motivated agent in McAdams (2016), but difficult to discern an integrative life story beneath it all.

Over the next 3 years, as I further researched Trump, I gradually came to realize that my inability to find evidence for a narrative identity in Trump’s life was less about the inadequacy of biographical sources, of which there are legion, and more about a bald psychological reality in Trump’s life. I became convinced that Trump has no narrative identity at all, or at best, he has a starkly depleted one. Trump does not see himself as a developing person who moves through time. The past has no purchase on him, and the future has no pull. Instead, he lives in the exuberantly combative moment, fighting like a boxer to win the round, fighting furiously as if it were the last round he will ever fight. The moments—the rounds, the episodes, the discrete scenes that would comprise a story if he had a life story—do not add up. They do not build in his mind to form a plot. The protagonist of this non-story never changes, never learns anything, never carries anything forward from one scene to the next. He is instead a “stable genius,” to quote one of Trump’s favorite self-attributions (Fritze & Collins, 2019). Like the main character in the movie *Memento*, Trump wakes up each morning with something akin to a blank slate. But unlike the movie, Trump does not suffer from retrograde amnesia or any other kind of purely cognitive deficit. This is not about dementia, for Trump has always lived his life this way, chosen to do so, it seems. He is capable of remembering yesterday, but yesterday is irrelevant except insofar that it can help him win today.

Let me be clear: I am not suggesting that Donald Trump has no sense of time’s passing. He grasps the temporality of life. He even understands the value of stories in general, as mechanisms for conveying meaning in time. His popular political slogan—“Make America Great Again”—suggests a culturally compelling story about the United States: Once upon a time, America was great; then it lost its greatness; and now it will recover the greatness it has lost. Moreover, Trump is perfectly capable of ascribing to himself a trait that might be central to the description of a character in a story. For example, he describes himself as a “fighter,” a “hero,” and so on. He has some degree of psychological insight, and he understands how others see him. But these self-attributions are always static rather than dynamic, as if he exists in the eternal present rather than in an unfolding narrative through which a protagonist *changes* or *develops* over time. Instead, his focus is mainly on the present episode within which he finds himself immersed, striving to triumph within that episode so that he can then move on to the next discrete episode.

Trump’s episodic approach to life frees him from the moral and normative conventions that constrain other human beings. It does not matter to Trump if what he says today blatantly contradicts what he said yesterday, or what he will say tomorrow. Critics can claim that Trump lies constantly (which is true), but “truth” for Trump is purely transactional, just like his relationships with people. What is true (or good) for Trump is what works to win the current episode. If saying “A” helps him win on Monday, then “A” is true. If saying “nonA” helps him win on Tuesday, then “nonA” is true. Both cannot be true, you say. But Trump does not consider the contradiction to be important; indeed, he may not even see the contradiction because for Trump, truth is episodic, as is life more generally.

Trump’s total embrace of the moment has always worked to his advantage, both in business and politics. For instance, his episodic nature gives him tremendous authenticity in the eyes of his millions of devoted fans. When they encounter Trump at a rally or watch him at a news conference, they know that he is ALL HERE NOW. He is not hiding anything. He is not planning the future or trying to stay consistent with the past. Even if every sentence that comes out of his mouth is a falsehood, he is telling it the way it is right now, in the moment, what he believes he needs to say in order to win the moment. It is shameless. It is primal. Unexpurgated, unmediated, completely divorced from doubt or reason or the need to be consistent and truthful in the long run, Trump erupts with what currently captures his consciousness, the unfiltered expression of his wholehearted embrace of the moment. Like an impulsive, angry child. Or a wild beast.

The argument I make in McAdams (2020) for the absence of a narrative identity in Trump’s personality makeup draws from many different incidents in his life and many different sources. Going back decades, journalists and biographers have expressed repeated frustration in their inability to elicit from Trump any kind of introspection or psychological commentary on his own life (e.g., D’Antonio, 2015; O’Brien, 2005). Trump talks about himself constantly, but never in narrative terms. Instead, he brags about achievements or proclaims his greatness. He attributes wonderful traits to himself—strength, courage, intelligence, power. He is a winner. He has never lost. He has never made a mistake. But for all his talk, Trump never delves beneath the surface; he rarely goes back in time; and he rarely projects very far into the future. He is not introspective; he is not retrospective; and he is not prospective. Unlike any president in modern times, he has virtually no sense of history, and he absolutely never talks about things like “posterity” or “legacy” or how “future generations” will look back upon the America of today. Donald Trump is no more able to speak in this exalted register—a favorite form of discourse for presidents like Ronald Reagan and Barack Obama—than he is to express empathy for the suffering of other people.

Writing for *The New Yorker* in the 1990s, Mark Singer spent a great deal of time with Trump urging him to reveal his inner self. What are you thinking about when you shave in the morning? Singer asked. What are the private feelings you keep to yourself? The questions made no sense to Trump. He could not answer them. Singer was forced to conclude that Mr. Trump has achieved something remarkable and utterly strange in human life: “an existence unmolested by the rumblings of a soul” (Singer, 2011). Tony Schwartz, who was Trump’s ghostwriter for his first book, *The Art of the Deal*, remarked that “Trump didn’t fit any model of human being I’d ever met.” He remembers almost nothing from his childhood, Schwartz said. “There is no private Trump” (Mayer, 2016). Reporting on Trump’s acceptance speech at the 2016 Republican National Convention, a *New York Times* journalist wrote: “After 40 years in the public eye, Mr. Trump decided on Thursday night that he was not interested in revealing himself to America with disarming tales of his upbringing, hard-earned lessons from his tumultuous career or the inner struggles masked by his outward pomposity.” In what was, at that point in his life, the most important speech he had ever given, Trump passed up the chance to “plumb his personal life and career for the kind of anecdotes that would turn him, in the eyes of his doubters, from a cartoon into a flesh-and-blood human being” (Barbaro, 2016).

Trump is not interested in casting himself as a flesh-and-blood human being. In his mind, he is more like a superhero. Shortly before he assumed the presidency, Trump told a group of advisors to think of everyday in the White House as a television show in which he vanquishes his rivals (Meacham, 2017). The sentiment is consistent with what Trump once described, in an interview for *People* magazine, as his philosophy of life: “Man is the most vicious of all animals, and life is a series of battles ending in victory or defeat” (D’Antonio, 2015, p. 154). For Trump, each day is a singular battle. But the successive battles do not build to form a readily defined war, the kind of war that has its own narrative arc, with clearly defined foes, stable alliances, clear issues of contention that drive antagonists apart, and the prospect that someday it will end—the war will be over, and we will look back on it and understand it as a story, as part of hi*story*. Instead, life is endless warfare, with no progression or direction. You fight furiously to win the day. You go to bed. And then you wake up to start it all over again. This is how Donald Trump has always lived—fully immersed in the combative here-and-now, living and fighting outside the flow of narrative time and history, the omnipotent I AM who never changes, never develops, but who simply IS.

## From Many Lives to One Life, and Back Again

I conceive of psychological biography as an effort to make psychological sense of an individual life through an artful application of sound scientific theories and validated empirical findings (McAdams, 2005). In the case of Donald Trump (McAdams, 2020), I drew heavily upon what psychological scientists have learned over the past few decades regarding the concepts of extraversion, agreeableness, narcissism, authoritarianism, cognitive styles, attachment patterns, leadership, and (especially) narrative identity, among other things. My reading of his life and his presidency led me to conclude that Trump lacks an inner story to provide his life with temporal continuity, purpose, and meaning. He is the episodic man, living (and fighting) in the moment. More than his socially dominant persona and more even than his raging narcissism, the strangest psychological feature of Donald Trump is his episodic manner of living and thinking outside the flow of narrative time.

Does Trump’s unique case suggest anything more general about human life? Ideally, science should progress through an ongoing dialectic between specific, concrete observations on the one hand and the generation and evaluation of more general, abstract conceptions on the other. In what Reichenbach (1938) called the *context of discovery*, scientists gather together observations in an effort to build a more general conception of how the world works. In psychology, case studies of individual lives hold great value for the generation of theory. They may yield new ideas whose applicability transcend the individual case itself. When it comes to psychological biography, the main purpose is nearly always to illuminate the single case at hand. But sometimes insights emerge that may hold broader significance or generalizability. Findings from the idiographic case study of a single life may prove to be useful for generating new hypotheses and ideas for nomothetic studies of many lives. These new ideas may transport themselves into Reichenbach’s (1938) *context of justification*, wherein they may be subjected to hypothesis-testing procedures and other scientific operations designed to articulate a more general understanding of the phenomena at hand. The results from hypothesis-testing studies may further modify and refine psychological theory, which eventually may be applied to new case studies which, in turn, may yield new and potentially generalizable insights. And the process continues.

Over the past two decades, empirical personality and developmental psychologists have built up and refined a broad set of theoretical formulations for understanding the structure, function, development, and psychological significance of narrative identity. With a few notable exceptions, however, they have tended to ignore the possibility that some people may have no narrative identity at all. The strange case of Donald Trump highlights the need to broaden theory in this domain and to launch new nomothetic studies, as well as further idiographic investigations, to examine what may be an important psychological difference between people. Empirical researchers have amply documented variations in the kinds of narrative identities people construct. Now they may wish to turn some attention to variability in the extent to which people construe their lives as narratives in the first place, and the psychological significance of this variability.

Initial forays into this new arena appeared even before the publication of McAdams (2020), suggesting that the idea may already be “in the air.” For example, one measurement-oriented research team has recently developed The Awareness of Narrative Identity Questionnaire (ANIQ: Hallford & Mellor, 2017). In this self-report instrument, research participants are asked to rate themselves on items like these: “When I think over my life, I can observe how there is a story that tells me who I am.” “Things that have happened over the course of my life are meaningfully tied together.” And, “There are clear themes relating to who I am that can be found in my personal memories.” The authors are approximating a dimension upon which “the episodic man” might characterize one manifestation of the low end, with the other extreme represented by a person who is acutely aware of and strongly involved in conceiving of life as a self-defining story with clear plot lines and integrative themes. In support of the validity of their measure, Hallford and Mellor (2017) report that participants who scored high on the ANIQ tended to describe “turning point” events in their lives that were judged by outside raters to be especially coherent, compared to individuals scoring lower on the ANIQ. It should be noted that other studies have shown that individuals diagnosed with borderline personality disorder (Adler, Chin, Kolisetty, & Oltmanns, 2012) tend to construct stories of personal experiences that are rated as lower on coherence, compared to matched controls. Other studies have shown a positive association between ratings of narrative coherence on the one hand and independent measures of mental health and well-being on the other (Baerger & McAdams, 1999; Waters & Fivush, 2015).

While low levels of narrative coherence may be part of the psychological picture for people who tend not to see their own lives in life-narrative terms, another component may be the relative absence of autobiographical reasoning. A distinguishing psychological feature of Donald Trump is his repeated refusal to derive broad conclusions about himself and his development from specific autobiographical memories. In *The Art of the Deal*, Trump (1987) does relate some memories from his early life. For example, he recalls punching his second-grade music teacher. Trump writes that the incident shows that he has always been a “fighter.” He remembers accompanying his father on trips through tough neighborhoods to collect rents from tenants. In one of these scenes, the young Donald asks Fred Trump why he stands to the side when he knocks on the door. Because sometimes they shoot right through the door, Fred Trump responds. The take-home message here is that you need to be careful because the world is a dangerous place. For the most part, the general conclusions drawn by Trump from his own memories reinforce his stated philosophy of life: “Man is the most vicious of all animals, and life is a series of battles ending in victory or defeat.” To the extent, then, that he engages in something like autobiographical reasoning, he does so either 1) to illustrate a trait (I am powerful, I am a fighter, I am smart) or 2) to illustrate a perceived truth about the world (danger, human viciousness).

A recent study in my lab illustrates a similar trend for a small minority of research participants who took part in life-story interviews (Turner, Cowan, Logan, & McAdams, in press). One of the main methods for collecting empirical data on narrative identity is a 2-hour life story interview, wherein research participants provide narrative accounts of important events in their lives as well as expected scenarios for the future (McAdams & Guo, 2015). In Turner et al. (in press), researchers administered self-report mood measures immediately before the interview began and at the end of the interview. For the sample of over 150 midlife adults who were interviewed, a strong positive emotional effect for the interview was observed. For the group as a whole, ratings on positive emotion increased significantly from before the interview to after. The finding confirms years of anecdotal data suggesting that most people very much enjoy telling their life stories. They find these interviews to be emotionally satisfying and psychologically fulfilling.

But a small group of participants (less than 10%) do not show the normative increase in positive emotion. Either their mood remains the same from before to after the interview, or else they decline somewhat in positive emotion. Turner and colleagues (in press) examined the interview transcripts for the 10 participants who most exemplified this trend, based on their self-reported emotion, and compared them to the 10 participants who showed the greatest increases in positive emotion. The biggest difference between the two groups concerned autobiographical reasoning. The 10 participants who did not find the life-story interview to be especially enjoyable rarely employed autobiographical reasoning in their interviews, even when asked to do so. For example, when the interviewer would ask them to spell out what they think a particular event in their lives “means” for their life story as a whole, or how the event “changed” them in any way, these participants tended to express frustration with the question, often saying that the event meant nothing at all, or that they did not know what the meaning was. When they did derive conclusions from the narrated events, they tended, like Trump, to say that the event illustrated an obvious trait in their lives (“It shows that I am a good person;” “It means that I enjoy parties”) or some perceived characteristic of life in general rather than their particular life (“It shows that life isn’t fair”).

The results from Turner et al. (in press) suggest that a relatively small minority of people do not resonate with the idea that life may be viewed as a story that provides a person with some degree of meaning and purpose. Instead, they may conceive of their lives in a more episodic manner, as a series of scenes that do not necessarily connect up with each other and that do not necessarily yield personal meaning. Future research needs to examine in more detail the episodic nature of some people’s self-conceptions. What does it feel like to experience life in an episodic manner? Are there psychological costs and benefits to construing life in this way? What are the developmental and cultural sources for the variation that people may show in the extent to which they operate as autobiographical authors?

## Conclusion

The prime goal in psychological biography is to make psychological sense of a particular person’s life. Employing various tools provided by the discipline of psychology, the biographer aims to enhance readers’ understanding of a specific human being. Therefore, a psychological biography of Donald Trump is successful, or not, mainly with respect to its effectiveness in promoting a better, deeper, or more adequate understanding of Donald Trump himself. In some cases, however, a psychological biography may offer the added value of suggesting an insight, idea, or hypothesis that may have more general significance, even to the point of informing subsequent empirical research and the further elaboration of psychological theory. By bringing to the fore Trump’s status as the episodic man, I believe that my psychological biography of the 45th president of the United States (McAdams, 2020) suggests new paths for empirical research in personality and developmental psychology, and perhaps other disciplines as well. The psychology of one particular human being—even a person as psychologically singular as Donald J. Trump—may sometimes hold implications for understanding the psychology of many.
